# The Effect of Injuries on Health Measured by Short Form 8 among a Large Cohort of Thai Adults

**DOI:** 10.1371/journal.pone.0088903

**Published:** 2014-02-13

**Authors:** Vasoontara Yiengprugsawan, Janneke Berecki-Gisolf, Roderick McClure, Matthew Kelly, Sam-ang Seubsman, Adrian C. Sleigh, the Thai Cohort Study Team

**Affiliations:** 1 National Centre for Epidemiology and Population Health, The Australian National University, Canberra, Australia; 2 Monash Injury Research Institute, Monash University, Melbourne, Australia; 3 School of Human Ecology, Sukhothai Thammathirat Open University, Nonthaburi, Thailand; CUNY, United States of America

## Abstract

**Introduction:**

We investigate the links between health and injury in Thailand. This is important because of the high burden of injury in transitional countries and limited information for public health.

**Methods:**

We analyse 2005 baseline and 2009, 4-year follow-up data from distance learning students of Sukhothai Thammathirat Open University residing nationwide (n = 60569). Injury was reported for the past year in both periods. Medical Outcome Study Short-Form (SF-8™) health status was reported and Physical and Mental Component Summary Scores (PCS and MCS) were calculated. Analyses used covariate-adjusted multivariate linear regression.

**Results:**

In 2009, increasing numbers of traffic injuries (0, 1, 2, 3, 4+) associated with declining PCS scores (49.8, 48.4, 46.9, 46.2, 44.0), along with a similar monotonic decline for MCS scores (47.6, 46.0, 44.2, 42.7, 40.6). A similar (but smaller) dose-response gradient was found between non-traffic injuries and SF-8 scores. Longitudinal analyses showed those with incident injury (no injury 2005, injury 2009) had lower PCS and MCS scores compared to those with no injury in both periods. Individuals with reverting injury status (injury 2005, no injury 2009) reported improvement in PCS and MCS scores over the four-year period.

**Conclusion:**

We found significant and epidemiologically important associations between increasing injury frequency and worse health in the past year, especially traffic injuries. Longitudinal 2005–2009 results were supportive and revealed statistically significant adverse 4-year effects of incident injury on health. If injury reverted over four years, low initial scores improved greatly. Findings highlight the importance of injury prevention as a public health priority.

## Introduction

Worldwide, injury has been shown to contribute a substantial and largely amenable component of the burden of morbidity and mortality. Indeed, fatal injuries cause some 10% of global mortality with the burden skewed toward the developing world. Some 90% of the global injury incidence is in the lower and middle-income countries, especially those with emerging economies and undergoing transition from infectious disease to chronic disease and injury [1]–[3]. In such settings, injury is among the top ten contributors to the overall burden of disease [4]–[8].

Prevention of injuries is now a public health priority [9]–[11]. But comprehensive injury prevention strategies in transitional developing country settings have not yet been defined. In such a transitional setting, injury prevention needs to be based on an understanding of the epidemiology and its consequences. Injury burdens differ among population subgroups; traffic or transport injuries are more common among young males and injuries at home, especially falls, affect older people [12]–[14]. An array of factors is known to increase injury risk including alcohol consumption, poor vision, obesity, and depression [14]–[18]. Some studies have reported that post-injury disabilities affect physical function and mental health and could result in long-term health impact [19]–[21]. But the consequences of non-fatal injuries on general health and quality of life remain largely unknown [22], [23].

In transitional Thailand, road traffic is the 3^rd^ highest cause of Disability Adjusted Life Years lost. Other types of injury including falls and self-harm are also in the top 20 causes [24]. Rates of all types of injury except assault have increased substantially in the last 20 years, especially falls and traffic injury which have both risen by more than 50% [25]. Also, as in many developing countries, traffic injury is the leading cause of death among young Thai males [24], [26], [27]. Occupational injury is also important with an estimated incidence rate of around 3–4% annually; however, official statistics from the Workers’ Compensation Fund underrepresent the real burden as they exclude workers in the informal sector who face greater risks [28], [29].

Our earlier reports examined injury frequency and determinants among a large cohort of Thai adults followed since 2005 [14], [30]–[32]. Here we continue our transitional setting analysis now moving down the causal pathway to focus on injury impact on physical and mental health in Thailand. We hypothesise that longitudinal analysis of health outcomes four years following injuries will reveal a close and coherent causal link. Such information on the health is needed to plan appropriate Thai programmes responding to injury.

## Methods

### Study population and data collection

This analysis is part of the overarching Thai Cohort Study (TCS), an ongoing epidemiological investigation of changing patterns of health-risk and health outcomes among Thai adults as they move away from traditional patterns of illness (maternal and child mortality and infectious diseases) to emerging chronic diseases and injury. Data derive from a research cohort of 87,134 distance-learning students aged between 15 and 87 years enrolled at Sukhothai Thammathirat Open University (STOU). The cohort members completed the first comprehensive mailed questionnaire and health survey in 2005. Four years later 40% were no longer enrolled because they had graduated or suspended their studies, however most continued to participate in the longitudinal assessments completed in 2009 (n = 60,569) and 2013 (underway).

The characteristics of cohort participants recapitulated well the distance learning student body at STOU and the general population of Thailand in which they are embedded [33], [34]. The similarity included their geographic distribution across Thailand and their modest incomes. At recruitment in 2005, there were slightly more females among the cohort, with the overall median age being 29 years. The 2005 baseline study and 2009 follow-up gathered data on a wide range of topics including socio-demo-geographic characteristics, health status, doctor diagnosed diseases, health service use, health-risk behaviours including smoking and drinking, as well as detailed injury-related questions. This report analyses the 2005 baseline and 2009, 4-year follow-up TCS data.

### Exposure, outcome, and confounders

In 2009, there were two sets of questions related to injuries: “In the last 12 months, how many times did you get injured in a traffic crash?” and “In the last 12 months, how many times did you have a non-traffic injury?” Response categories for each question include: ‘never’, ‘one’, ‘two’, ‘three’, ‘four or more’. For each injury type, two additional questions were asked: “When you experienced your most serious (traffic or non-traffic) injury, did you receive medical care [yes or no]?” “Did this most serious (traffic or non-traffic) related injury limit your normal activities for one day or more [yes or no]?” For traffic injury, data on role during the injury (driver, passenger, pedestrian) and types of vehicle (bicycle, motorcycle, car, etc.) were also collected. For non-traffic injury, location (home, sport, or workplace) and nature of injuries (fracture, sprain, cut, bruise, burn, concussion, or internal injury) were included.

Measured outcomes in 2009 were the Medical Outcome Study Short-Form (MOS SF-8™) Health Survey which includes one question with ordinal outcome categories on each of the following eight domains: general health, physical functioning, role physical, bodily pain, vitality, social functioning, mental health, and role emotional. All categorical responses for each domain are numerically scaled according to the SF-8 international standard [35]. Higher scores indicate better status. To compute summary scores, international physical or mental weights were applied to each domain value before all eight domains were summed and a physical or mental constant added [35]. The resulting Physical Component Summary (PCS) and Mental Component Summary (MCS) scores are designed for a normal population to average a value of 50 and standard deviation of 10 (norm-based standardization). The SF measures have been extensively used to assess health and quality of life, including after injuries [36]–[38].

Potential confounders included in this analysis have been found to associate with self-assessed health among cohort members [39], [40]. They include age-sex categories (male or female, <40 or ≥40 years), household monthly income categories, and residence (rural, urban), health-risk behaviour (smoking and alcohol drinking), and self-report of doctor-diagnosed chronic metabolic or cardiovascular disorders (e.g. diabetes, hypertension).

### Data processing and statistical analysis

Questionnaire responses were optically digitized and subsequently edited used Thai Scandevet, SQL and SPSS software. For analysis we used Stata version 12. Individuals with missing data for given analyses were excluded so totals vary slightly according to available information. Statistically significant level was set at p<0.05 and unless otherwise indicated differences between groups are generally significant due to a large sample.

Injury over the 12-month period preceding the 2009 questionnaire was considered the ‘exposure’ and SF-8 PCS and MCS scores over the last four weeks were the ‘health outcomes’. Unadjusted mean PCS and MCS scores were reported by number of injuries, calculated separately for each age-sex category. We then used multivariate linear regression estimating associations between the number of traffic and non-traffic injuries (in the past 12 months) and mean PCS and MCS scores (in the last four weeks), adjusting for potential confounders.

The analysis then proceeded to use the longitudinal 2005–2009 data as follows. In 2005, most of the injury-related questions were the same as in 2009 with the exception of the overall injury question which added the italicized words that follow: “In the last 12 months how many serious injuries have you have had that *were enough to interfere with daily activities and/or required medical treatment*?” The 2005–2009 longitudinal analyses were restricted to injuries that were serious enough to interfere with daily activities and/or required medical treatment in both periods. This restriction was introduced so that the injury data gathered in the two surveys would be harmonious. Cohort members fell into four exposure groups according to their 2005 and 2009 injury status: ‘null’ (no injury in both periods), ‘reverting’ (injury reported for 2005 but not for 2009), ‘incident’ (no injury reported for 2005 but injury reported for 2009), and ‘chronic’ (injury reported for both periods). For the 4-year changes in mean outcome scores, we calculated the difference in PCS and MCS scores for each individual, subtracting 2005 values from the 2009 values. Therefore, if scores got worse over time the score difference would be negative, and vice versa.

Next we proceed to the final longitudinal analyses using multivariate linear regression providing valid measures of effects of injury on health. These measures involve calculating adjusted mean SF-8 scores at the starting point (2005 baseline) and longitudinal 2005–2009 adjusted SF-8 score differences, correcting for the influence of confounding variables (age-sex and household income categories, residence, health-risk behaviours, and doctor diagnosed chronic conditions). These longitudinal results are compared for the four injury exposure groups (null, reverting, incident, and chronic).

### Ethical considerations

Informed written consent was obtained from all participants. Ethics approval was obtained from Sukhothai Thammathirat Open University Research and Development Institute (protocol 0522/10) and the Australian National University Human Research Ethics Committee (protocols 2004/344 and 2009/570). All students were advised that they could withdraw, or not participate, without any effect on their academic progress. They were assured that their information was confidential and their identity would never be revealed. The questionnaires never sought sensitive personal information and no biological samples were taken. Data were anonymized before analysis. No children and minors were recruited to this study; however, a small number of 15–17 year olds (<0.1%) enrolled at STOU responded to the baseline questionnaire. These adolescents were enrolled in a program for gifted young people with agreement between the university and each of their guardians. The ethics committees provided approval for recruiting all Open University students. In addition, we contacted by phone the guardians of respondents aged 15 to 17 at the 2005 baseline to verify participants ages and to obtain additional verbal consent. It is important to note that data used in this study were based on 2009 follow-up questionnaire, which was collected 4 years after the 2005 baseline questionnaire. Hence we can confirm that all respondents were aged over 18 years in our data presented here.

## Results

There were 60569 cohort members in 2009; dividing by age (< and ≥40 years) the cohort included 26.7% younger males, 39.9% younger females, 18.6% older males, and 14.9% older females. Approximately 10% of cohort members reported at least one traffic injury in the past year (Table 1). There were 4.3% of cohort members who reported traffic injuries which limited activities for at least one day. When asked about their role during the injury, 7.1% reported being a driver (10.6% among young males vs 3.7% among older females). The most commonly reported vehicles used when injured were motorbikes and this was most common among younger cohort members (8.8% among young males vs 6.7% among young females, 4.8% among older males and 6.7% among older females).

**Table 1:** Distribution of cohort attributes by age and sex, Thai Cohort Study 2009.

Cohort attributes	Overall %	Age-sex categories (column %)			
		< 40 years		≥40 years	
		Male (n = 16165)	Female (n = 24165)	Male (n = 11242)	Female (n = 8997)
***Monthly household income (Baht)***					
≤10000	21.2	25.0	25.3	14.2	12.0
10001–20000	23.6	27.4	26.8	17.2	16.5
20001–30000	21.3	22.8	20.6	21.9	20.0
30001–50000	17.8	13.7	14.9	24.2	25.2
>50000	13.2	8.2	10.2	19.1	22.8
Not answered	2.9	3.0	2.2	3.5	3.7
***Residence***					
Rural	44.0	47.5	44.7	44.0	36.0
Urban	56.0	52.5	55.4	56.0	64.0
***Smoking***					
Never	76.8	58.7	96.8	44.1	96.4
Former	8.9	19.2	0.7	18.2	0.8
Regular	14.3	22.1	2.5	37.7	2.8
***Drinking***					
Never	72.0	48.5	89.9	51.5	91.9
Social	20.9	36.6	9.1	34.4	7.4
Regular	7.1	14.9	1.0	14.1	0.7
**Chronic conditions**					
Yes	18.6	12.8	7.9	42.3	28.5

Non-traffic injury was also common among cohort members (12.8% reported once 8.1% reported twice, 6.4% reported three times, and 7.8% reported four times or more). There were 8.9% of cohort members who reported non-traffic injuries which limited activities for at least one day. Regarding location of occurrence of reported injuries, 13.5% reported at home (9.7% among males and 16.5% among females). Injuries at sport facilities were more common among younger males. Male cohort members reported injuries of a more severe nature: fracture, concussion, and internal injuries. Female cohort members suffered more bruises than their male counterparts (16.9% among younger females vs 13.4% among younger males and 12.7% among older females vs 9.6% among older males).

In addition to different injury patterns among age-sex categories, there are certain cohort attributes which are distributed differently across age-sex groups (Table 2). For example, chronic conditions were almost three times more prevalent among older cohort members. Older cohort members also tend to have higher household incomes and urban females were generally older. With regard to health-risk behaviours, smoking was much more prevalent among males (22.1% vs 2.5% in younger and 37.7% vs 2.8% among older cohort members). Similarly, alcohol consumption was more common among males.

**Table 2:** Distribution of injuries in the past year by age and sex, Thai Cohort Study 2009.

Types of injuries	Overall %	Age-sex categories (column %)			
		< 40 years		≥40 years	
		Male (n = 16165)	Female (n = 24165)	Male (n = 11242)	Female (n = 8997)
***Overall traffic injuries***					
Traffic-injury by frequency	9.9 (5608)	12.7	9.8	7.6	6.6
One	7.7 (4390)	9.4	7.9	6.2	5.6
Two	1.5 (834)	2.1	1.4	1.0	0.8
Three	0.5 (270)	0.8	0.4	0.3	0.2
Four or more	0.2 (114)	0.4	0.1	0.14	0.05
Medical care required					
Yes	4.0 (2440)	5.6	3.6	3.7	2.7
Injury limit activities ≥1 day					
Yes	4.3 (2611)	6.0	4.1	3.7	2.5
Role during the injuries*					
Driver	7.1 (4272)	10.6	6.3	6.4	3.7
Passenger	1.8 (1083)	1.2	2.7	0.7	1.9
Pedestrian	0.4 (250)	0.4	0.5	0.3	0.5
Type of vehicle*					
Bicycle	0.8 (511)	1.2	0.8	0.8	0.5
Motorbike	6.5 (3911)	8.8	6.7	4.8	3.6
Car	1.0 (629)	1.4	0.8	1.3	0.8
Bus, van, coach	0.3 (193)	0.2	0.4	0.1	0.6
***Overall non- traffic injuries***					
Non-traffic injury by frequency	35.1(20014)	38.8	35.9	30.8	31.2
One	12.8 (7335)	13.9	11.4	13.7	13.7
Two	8.1 (4602)	8.5	8.4	7.5	7.1
Three	6.4 (3641)	7.1	6.8	5.1	5.5
Four or more	7.8 (4436)	9.3	9.3	4.5	4.9
Medical care required					
Yes	8.0 (4823)	9.8	7.1	7.4	7.7
Injury limit activities ≥1 day					
Yes	8.9 (5417)	11.4	8.3	8.0	7.5
Location of injuries					
Home	13.5 (8145)	9.7	16.8	9.7	16.1
Sport facility	3.7 (2223)	7.6	1.4	4.9	1.0
Workplace (agriculture)	3.0 (1829)	4.7	1.9	4.2	1.5
Workplace (non-agriculture)	7.2 (4362)	9.3	7.7	5.1	4.8
Nature of injuries*					
Fracture	1.1 (647)	1.6	0.6	1.2	1.1
Sprain/ strain/ dislocation	10.9 (6586)	13.7	9.8	10.8	8.7
Cut/ bite/ open wound	11.7 (7084)	13.6	12.6	9.5	8.5
Bruise	14.0 (8467)	13.4	16.9	9.6	12.7
Burn/scald	2.1 (1270)	1.6	2.8	1.1	2.4
Concussion	4.6 (2780)	6.1	4.4	3.9	3.3
Internal injury	1.9 (1147)	2.2	1.7	2.0	1.8

*multiple responses were allowed.

In Table 3, we break down reported traffic and non-traffic injuries (once, ≥ twice in the past year) by age-sex categories, income, rurality, smoking, drinking, and self-report of metabolic or cardiovascular conditions. Generally in both categories of reported injury frequency, males had higher proportions of traffic injuries. Overall there is an inverse association between increasing household income and decreasing proportions, with some exceptions among highest income group reporting non-traffic injuries. Compared to ‘never’ smokers or ‘never’ drinkers, regular smoking and regular alcohol drinking were associated with higher proportions reporting traffic or non-traffic injuries.

**Table 3:** Age-sex-specific traffic and non-traffic injury rates by personal attributes, Thai Cohort Study 2009.

Number of injuries (once, twice) by personal attributes	Traffic injuries				Non-traffic injuries			
	< 40 years		≥40 years		< 40 years		≥40 years	
	Male	Female	Male	Female	Male	Female	Male	Female
***Reported injury once in the past year***								
**Monthly household income**								
≤10000 Baht	11.4	10.1	9.8	8.4	14.3	11.9	16.1	16.1
10001–20000 Baht	10.0	8.2	8.2	6.7	14.3	11.1	13.6	13.3
20001–30000 Baht	9.4	7.6	6.2	6.6	13.4	10.8	13.9	13.9
30001–50000 Baht	7.4	5.9	4.9	4.2	13.1	12.2	13.2	13.0
>50000 Baht	6.2	5.4	3.8	4.0	14.0	11.6	14.5	13.0
**Residence**								
Rural	9.8	8.5	6.1	5.6	13.0	14.1	11.7	13.4
Urban	9.1	7.4	6.3	5.4	12.7	13.7	11.2	13.9
**Smoking**								
Never	8.6	7.7	5.6	5.4	13.6	11.4	13.3	13.5
Former	12.1	12.9	8.2	11.5	14.2	14.9	14.0	21.0
Regular	9.6	13.8	5.9	9.7	14.8	13.5	13.8	15.6
**Drinking**								
Never	7.3	7.5	5.1	5.3	13.0	11.4	12.6	13.5
Social	10.5	10.4	6.6	5.4	14.4	10.8	15.2	14.4
Regular	12.9	16.7	8.6	7.4	15.7	12.3	14.3	15.8
**Chronic conditions**								
No	9.6	8.0	6.3	5.6	14.0	11.4	13.3	13.4
Yes	8.2	7.0	6.0	5.6	13.5	11.8	16.8	14.4
***Reported injury ≥twice in the past year***								
**Monthly household income**								
≤10000 Baht	5.0	3.0	2.3	3.0	28.1	25.5	18.5	18.1
10001–20000 Baht	2.9	2.0	2.6	1.5	24.6	24.3	18.0	17.1
20001–30000 Baht	2.6	1.6	1.4	1.3	23.8	23.6	17.0	17.9
30001–50000 Baht	2.6	1.4	1.0	0.6	22.2	22.8	17.0	17.0
>50000 Baht	2.8	1.4	0.9	0.2	25.5	26.5	16.6	18.4
**Residence**								
Rural	3.6	2.1	1.5	1.2	22.8	26.0	24.3	18.1
Urban	3.1	1.9	1.5	1.1	21.8	24.0	24.6	16.3
**Smoking**								
Never	2.6	1.8	1.2	1.0	23.8	24.1	16.0	17.2
Former	5.5	5.5	2.6	4.9	27.4	30.4	18.0	24.2
Regular	3.5	5.4	1.4	0.9	26.0	37.1	18.1	27.2
**Drinking**								
Never	2.2	1.7	1.2	1.0	24.4	24.0	16.1	17.2
Social	4.0	3.4	1.6	1.6	25.9	29.8	18.0	19.3
Regular	5.6	5.9	2.2	5.3	25.6	33.6	19.7	21.2
**Chronic conditions**								
No	3.5	2.0	1.6	1.1	25.0	24.1	14.2	16.8
Yes	2.4	2.1	1.5	1.1	24.3	28.0	17.5	19.4

In Table 4, we report the 2009 association between number of injuries and PCS and MCS scores using multivariate linear regression, adjusting for cohort attributes as reported in Table 2. For both traffic and non-traffic injuries, we found a monotonic dose-response gradient between increasing number of injuries and decreasing PCS and MCS scores but we note that the magnitude of the gradient was much larger for traffic injuries. An increasing number of traffic injuries (0, 1, 2, 3, 4+) was associated statistically (p<0.05) with declining PCS mean scores (49.8, 48.4, 46.9, 46.2, 44.0), with a similar monotonic decline for MCS scores (47.6, 46.0, 44.2, 42.7, 40.6). A similar dose-response gradient but of smaller magnitude was found between increasing number of non-traffic injuries and decreasing mean PCS and MCS scores.

**Table 4:** Association between injury and SF-8 health scores, Thai Cohort Study 2009.

Frequency of injuries	Adjusted SF-8 health scores [95% Confidence Intervals]*			
	Physical Component Summary (PCS)		Mental Component Summary (MCS)	
	Score in 2009	Score deviance**	Score in 2009	Score deviance**
***Traffic injury***				
Never	**49.8** [49.5–50.0]	reference	**47.6** [47.3–47.9]	reference
One	**48.4** [48.2–48.6]	–1.39 [–1.61, –1.16]	**46.0** [45.7–46.3]	–1.59 [–1.86, –1.32]
Two	**46.9** [46.4–47.4]	–2.89 [–3.39, –2.39]	**44.2** [43.6–44.9]	–3.35 [–3.96, –2.74]
Three	**46.2** [45.4–47.1]	–3.55 [–4.43, –2.68]	**42.7** [41.7–43.8]	–4.88 [–5.95, –3.82]
Four or more	**44.0** [43.1–45.7]	–5.42 [–6.73, –4.12]	**40.6** [39.0–42.2]	–6.98 [–8.57, –5.40]
***Non-traffic injury***				
Never	**50.2** [50.0–50.5]	reference	**48.1** [47.8–48.4]	reference
One	**48.8** [48.7–49.0]	–1.37 [–1.55, –1.19]	**46.8** [46.6–47.0]	–1.24 [–1.46, –1.03]
Two	**48.5** [48.3–48.7]	–1.72 [–1.94, –1.50]	**46.1** [45.9–46.4]	–1.93 [–2.19, –1.66]
Three	**48.5** [48.3–48.7]	–1.71 [–1.95, –1.46]	**45.8** [45.5–46.1]	–2.22 [–2.52, –1.93]
Four or more	**47.8** [47.6–48.1]	–2.35 [–2.58, –2.13]	**45.1** [44.9–45.4]	–2.92 [–3.19, –2.65]

* Physical and Mental Component Summary scores were produced by multivariate linear regression fitting injury frequency to SF-8 summary health outcomes after adjusting for age-sex categories, household income, urban-rural residence, health-risk behaviours (smoking, alcohol drinking), and doctor-reported chronic conditions.

** Score differences for each injury frequency express the deviance from the reference scores for the ‘never’ group. For example, cohort members with one traffic injury reported a PCS score of 48.4, or 1.39 lower than the ‘never’ group (49.8).

The longitudinal 2005–2009 injury data revealed that about 72% of cohort members had no injury in both periods, almost 12% reported ‘reverting’ injury status (injury for 2005 but not for 2009), about 12% of cohort members had ‘incident’ injury status (no injury for 2005 but injury for 2009), and less than 5% of cohort members reported injury in both periods (Table 5). The principal longitudinal results are derived from covariate-adjusted multivariable linear regression on baseline scores (2005) and 4-year score differences (2005 to 2009). Full longitudinal analyses (2005–2009) revealed that individuals reporting injury in both periods had the lowest baseline health scores and the smallest longitudinal health score changes which were not statistically significant. Those with 4-year ‘incident’ injury status had the largest adverse change in their PCS (–1.39) and MCS (–1.02) scores. Cohort members with ‘reverting’ injury status had the largest PCS (1.73) and MCS (1.16) score improvement over the four-year period. Cohort members reporting baseline injury (i.e. ‘yes-no’ and ‘yes-yes’ over the four-year period) began with lower PCS and MCS scores in 2005 than the other two groups; also their (small) 4-year longitudinal changes were statistically significantly different (p<0.05) from cohort members with no injury at both periods.

**Table 5:** Longitudinal association between injury status and SF-8 health, Thai Cohort Study 2005–2009.

Longitudinal injury status* (%, n)	Four-year change in adjusted SF-8 scores [95% Confidence Intervals]	
	Physical Component Summary scores	
	Score in 2005**	Longitudinal score change***
2005-no 2009-no (72.1, 39708)	50.2 [49.8–50.5]	–0.90 [–1.23, –0.58]
2005-yes 2009-no (11.9, 6569)	47.4 [46.3–48.4]	1.73 [1.50, 1.96]
2005-no 2009-yes (11.7, 6428)	49.2 [48.3–50.1]	–1.39 [–1.63, –1.17]
2005-yes 2009-yes (4.4, 2399)	46.3 [44.6–48.1]	–0.05 [–0.42, 0.31]
	**Mental Component Summary scores**	
	**Score in 2005** **	**Longitudinal score change** ***
2005-no 2009-no	48.0 [47.6–48.4]	–0.37 [–0.76, 0.01]
2005-yes 2009- no	46.0 [44.8–47.2]	1.16 [0.89, 1.42]
2005-no 2009- yes	47.1 [46.0–48.2]	–1.02 [–1.29, –0.75]
2005-yes 2009- yes	44.9 [42.8–46.8]	0.18 [–0.16, 0.51]

* Longitudinal analyses were restricted to injuries serious enough to interfere with daily activities and/or require medical treatment.

** Baseline scores were calculated from multivariate linear regressions adjusted for 2005 covariates including age-sex categories, household income, residence, smoking, alcohol drinking, and one of the doctor-reported chronic conditions.

*** Score changes were calculated for each individual by subtracting the 2005 scores from 2009 scores. Then, multivariate linear regressions of score changes as a function of longitudinal injury categories were adjusted for 2009 covariates including age-sex categories, household income, residence, smoking, alcohol drinking, and one of doctor-reported chronic conditions.

## Discussion

Injuries were a common event amongst our adult Thai cohort study members. Traffic injuries were more frequent among younger individuals. Non-traffic injuries were also very common with 27% reporting up to three injuries and 8% reporting more than four injuries in the past year. Home injuries occurred more often among females and sporting injuries were common among males. When we examined the 2009 association between number of injuries in the past year and physical and mental health in the past month, we found a strong connection. As the number of injuries progressively increases, there is a monotonic fall in both physical and mental health; these 2009 relationships were unequivocal and substantial and much larger in magnitude for traffic compared to non-traffic injuries. Furthermore, 4-year longitudinal results showed that cohort members with ‘incident’ injury status had substantial deterioration in their health. In sharp contrast, cohort members with ‘reverting’ injury status reported better health with both PCS and MCS scores improving over the four years following the 2005 injury. The ‘chronic’ injury status group had the lowest baseline health scores and the least improvement.

Our study found the magnitude of physical and mental health impacts from traffic injuries to be substantial and significant; this finding supports previous studies that showed injuries impact on physical and mental health based on quality of life measures [22], [23]. One prospective cohort study using SF-36 showed worse post-injury scores, especially for mental health [41]. Another study reported the impact of back, head, and neck injuries on self-assessed physical and mental health which was significant up to 10 years after the event [19]. In our cohort, despite a smaller magnitude epidemiological effect when compared to traffic injuries, the prevalence of non-traffic injuries in the past year was high. Other studies have reported non-traffic injuries such as falls to be associated with poorer health and lower quality of life [42], [43].

The strength of our cohort lies in the large study population of Thai adults and the comprehensive questions on injury, socio-demo-geographic attributes, health behaviours, and health outcomes. This is valuable additional information as most injury data in developing countries derive from hospital records which only include injuries resulting in medical treatments [44], [45]. We note that the tertiary education level of participants helps accurate completion of comprehensive questionnaires including self-reported accuracy of injuries However, there could be potential bias compared with the general Thai population with lower education level.

Aware of potential recall bias of self-reported injury in our study, we restricted initial analyses to 2009 follow-up data. We set the recall period for injury at 12 months and the recall period for SF-8 physical and mental health is always past four weeks. It is certain that most analysed injuries would have occurred distributed over 12 months and would have preceded health outcomes reported. This internal 2009 longitudinal dimension was expanded when we formally conducted a 2005–2009 longitudinal analysis connecting the 2005 injury exposure data with 4-year 2009 health outcomes. The results by both methods were in agreement which is evidence of study validity. We acknowledge that our study was based on injury data at two time-points (2005 and 2009) and there could be other injuries occurring between those four years which were not captured.

This is one of the first studies to investigate the association between injuries and health in middle-income Southeast Asian countries. In the 2009 assessment, there were statistically significant and epidemiologically important associations between increasing injury frequency and worse health, especially for traffic injuries. The longitudinal 2005–2009 results were supportive and revealed statistically significant adverse 4-year effects of incident injury on health. If injury reverted over four years, the low initial scores improved greatly. If injury was reported in both 2005 and 2009, the adverse health effects (low SF-8 score) were detected in both years.

Our study is large and longitudinal and we adjusted for confounders and used standard instruments to measure holistic health outcomes. So we are confident that the injury-health relationships revealed are substantive and causal. Injury prevention in Thailand should lead to a prompt improvement in both physical and mental health. We have also shown that SF-8 measurements produce standardised coherent information on health outcomes and can detect and quantify injury impacts at the population level.
